# Mobile Diary App Versus Paper-Based Diary Cards for Patients With Borderline Personality Disorder: Economic Evaluation

**DOI:** 10.2196/28874

**Published:** 2021-11-11

**Authors:** Sidsel Lund Laursen, Stig Helweg-Jørgensen, Astrid Langergaard, Jesper Søndergaard, Sabrina Storgaard Sørensen, Kim Mathiasen, Mia Beck Lichtenstein, Lars Holger Ehlers

**Affiliations:** 1 Department of Clinical Medicine Danish Center for Healthcare Improvements Aalborg University Aalborg Oest Denmark; 2 Research Unit for Telepsychiatry and E-mental Health Mental Health Services in the Region of Southern Denmark Odense Denmark; 3 Department of Clinical Research University of Southern Denmark Odense Denmark

**Keywords:** borderline personality disorder, dialectical behavior therapy, mobile app, psychotherapy, cost-consequence, mHealth, mobile phone

## Abstract

**Background:**

The cost-effectiveness of using a mobile diary app as an adjunct in dialectical behavior therapy (DBT) in patients with borderline personality disorder is unknown.

**Objective:**

This study aims to perform an economic evaluation of a mobile diary app compared with paper-based diary cards in DBT treatment for patients with borderline personality disorder in a psychiatric outpatient facility.

**Methods:**

This study was conducted alongside a pragmatic, multicenter, randomized controlled trial. The participants were recruited at 5 Danish psychiatric outpatient facilities and were randomized to register the emotions, urges, and skills used in a mobile diary app or on paper-based diary cards. The participants in both groups received DBT delivered by the therapists. A cost-consequence analysis with a time horizon of 12 months was performed. Consequences included quality-adjusted life years (QALYs), depression severity, borderline severity, suicidal behavior, health care use, treatment compliance, and system usability. All relevant costs were included. Focus group interviews were conducted with patients, therapists, researchers, and industry representatives to discuss the potential advantages and disadvantages of using a mobile diary app.

**Results:**

A total of 78 participants were included in the analysis. An insignificantly higher number of participants in the paper group dropped out before the start of treatment (*P*=.07). Of those starting treatment, participants in the app group had an average of 37.1 (SE 27.55) more days of treatment and recorded an average of 3.16 (SE 5.10) more skills per week than participants in the paper group. Participants in both groups had a QALY gain and a decrease in depression severity, borderline severity, and suicidal behavior. Significant differences were found in favor of the paper group for both QALY gain (adjusted difference −0.054; SE 0.03) and reduction in depression severity (adjusted difference −1.11; SE 1.57). The between-group difference in total costs ranged from US $107.37 to US $322.10 per participant during the 12 months. The use of services in the health care sector was similar across both time points and groups (difference: psychiatric hospitalization <5 and <5; general practice −1.32; SE 3.68 and 2.02; SE 3.19). Overall, the patients showed high acceptability and considered the app as being easy to use. Therapists worried about potential negative influences on the therapist-patient interaction from new work tasks accompanying the introduction of the new technology but pointed at innovation potential from digital database registrations.

**Conclusions:**

This study suggests both positive and negative consequences of mobile diary apps as adjuncts to DBT compared with paper diary cards. More research is needed to draw conclusions regarding its cost-effectiveness.

**Trial Registration:**

ClinicalTrials.gov NCT03191565; http://clinicaltrials.gov/ct2/show/NCT03191565

**International Registered Report Identifier (IRRID):**

RR2-10.2196/17737

## Introduction

### Background

Borderline personality disorder (BPD) is estimated to affect between 0.7% and 4.8% of the general population in Scandinavia and accounts for approximately 15% of adult admissions in Danish psychiatric hospitals [1-3]. Patients diagnosed with BPD are often characterized as emotionally unstable, impulsive, and self-harming and have unstable relationships [4]. It has been shown that the mortality rate is 8 times higher in patients with BPD than in the general population [5]. Furthermore, BPD has large societal consequences, as one study has found that the total mean societal cost related to BPD is US $32,863.01 per patient per year [6].

One of the most well-researched and clinically effective psychosocial treatments for patients with BPD is dialectical behavior therapy (DBT) [7-12]. Studies have shown DBT to be effective in reducing nonsuicidal self-injury and increasing quality-adjusted life years (QALYs), when compared with treatment as usual or client-centered therapy [13-16]. The DBT includes elements from cognitive-behavioral therapy and is centered on learning a set of predefined skills, such as mindfulness, tolerance of distress, regulation of emotions, and navigation of interpersonal situations [17,18]. Learning the DBT skills is done through self-monitoring using weekly paper-based diary cards filled out by the patient [19,20]. The use of paper-based diary cards, however, has limitations for both patients and therapists. Working with paper diaries is burdensome and may reduce patient engagement in treatment [21,22]. Retrieving and reviewing paper diaries to evaluate the patient’s progress over time is a time-consuming process for the therapist, and it is not possible for the therapist to follow the patient alongside self-treatment at home [20-22].

New studies suggest that mobile diary apps can overcome some of these limitations and be more effective in reducing distress symptoms and suicidal and nonsuicidal self-harming behaviors compared with treatment with DBT alone [23-25]. Mobile apps have the potential to reduce barriers and increase engagement in treatment [21,22]. Mobile apps can automatically provide an overview of the patients' progress and enable therapists to monitor the present emotional stage of patients during treatment [21,24,26].

### Objective

Economic evaluations of new health technology constitute important knowledge for decision-makers in health care as they estimate the value for money of the technology compared with alternative use of the scarce financial resources. To our knowledge, no economic evaluation has yet been conducted on the use of mobile apps as adjuncts to DBT in patients with BPD. Therefore, the aim of this economic evaluation is to investigate the costs and consequences of a mobile diary app compared with paper-based diary cards for patients with BPD, within the context of a psychiatric outpatient facility.

## Methods

### Overview

This economic evaluation was reported in line with the Consolidated Health Economic Evaluation Reporting Standards [27]. It was conducted alongside a pragmatic, multicenter, randomized controlled trial as part of the ENTER program [28]. The clinical study is described in detail in the study protocol [29] but will be summarized in the Methods section of this evaluation. Economic evaluation was carried out as a cost-consequence analysis, which is why costs and consequences are shown separately [30]. The cost-consequence analysis approach was chosen because of the possibility of investigating a broad range of consequences [30], such as intermediate outcomes and qualitative aspects. The time horizon was restricted to 12 months, and all relevant costs were included in the analysis. All costs were measured in Danish Krone (DKK) and subsequently converted to the US dollars (US $).

### Participants

The participants were recruited from 5 Danish psychiatric outpatient facilities between June 2017 and December 2018. The participants were eligible for inclusion if they were ≥18 years of age, formally diagnosed by a psychiatric specialist with Emotionally Unstable Personality Disorder (F60.3 according to ICD-10 criteria) [31], and admitted for psychiatric outpatient treatment. To be included, participants also had to be either self-harming or to have had suicidal behavior within the last 3 years. Participants with comorbid disorders, such as depression, anxiety, and posttraumatic stress disorder, were allowed to participate. On the contrary, participants were excluded if they had no access to a working smartphone; an IQ <70; or a comorbid disorder such as substance abuse, bipolar disorder, or a disorder within the schizophrenic spectrum. Before entering the study, all participants signed an informed consent form.

### Interventions

Participants were randomly assigned to either the mobile diary app group or to the paper-based diary card group using REDCap (Research Electronic Data Capture) [29]. In both groups, participants had to register entries about aspects such as emotional dysregulation, suicidal and self-harm thoughts, and skill use. The participants received the paper-based treatment registered daily on paper diary cards, whereas the participants using the mobile diary app were registered daily via the mDiary app (The Monsenso system). The mDiary app is a market solution containing psychoeducational material and visualizations of the participant’s data, which can be used in therapy sessions and for real time monitoring alongside treatment [32]. Participants in both groups received standard DBT (delivered as individual sessions, group sessions, and telephone coaching). The therapists instructed the participants on how to use the app or paper diary cards. Other treatments, such as medications, were allowed. Passive sensor data (eg, activity level and phone use) were collected from all participants through their smartphones. The treatment period was 40 weeks at 2 sites and 12 months at the other sites, and neither the participants nor the therapists were blinded to the group allocation.

### Consequences

#### Health-Related Outcomes

Health-related quality of life was based on the EuroQol 5-Dimensions 5-Levels (EQ-5D-5L) questionnaire [33]; depression severity from the Patient Health Questionnaire-9 [34]; borderline severity was assessed using the Zanarini Rating Scale for BPD [35]; and suicide behavior from the Suicide Behaviors Questionnaire [36]. Data were collected at baseline and at the 12-month follow-up. The participants' utility scores were calculated using the Danish weights for the EQ-5D-5L questionnaire [37]. QALY gain was estimated by assuming linear interpolation between utility scores at baseline and a 12-month follow-up, after which the area under the curve was calculated [38]. For depression severity, borderline severity, and suicide behavior, the difference from baseline to follow-up was calculated and compared between the groups.

#### Health Care Sector

In Denmark, the civil registration number makes it possible to link information from several registries to an individual [39]. Information on psychiatric hospitalizations and consultations with general practice was obtained at an individual level from the Primary National Health Insurance Service Register and the Danish National Patient Registry [40,41]. The specific purpose of the consultations in general practice was not available, which is why all consultations were included. The number of psychiatric hospitalizations and consultations with general practice was estimated 30 days before and after baseline and 12 months before and after baseline, respectively.

#### Treatment Compliance and Skill Recordings

It was continuously assessed whether the participants were still receiving treatment and whether they were recording their skills use. The recording of the skills used per week was estimated at an individual level as the total number of skills for each participant divided by the number of days in treatment, which was subsequently calculated for a week. A participating therapist estimated the time spent on recording in the 2 groups based on experience from the trial.

*The usability* of the mobile diary app was assessed by patients and therapists using a system usability scale [42]. This assessment was conducted in focus group interviews alongside a feasibility study of the app.

*Future innovation potential* was assessed by app developers, researchers, and other personnel related to the study. This assessment was conducted during a workshop to discuss the advantages, disadvantages, and development potential.

### Costs

All relevant costs were included in the analysis and adjusted to the price level in 2019 using the Danish net price index [43]. The conversion rate from kr to US $ was kr 634.0 per US $100 from September 23, 2021 [44]. The costs were not discounted because of a time horizon of 12 months [45].

The costs of health care services, patient costs, and municipality costs were used to adjust for baseline differences in the analysis. Health care services included primary care services, prescription medicine, inpatient and outpatient services, and emergency contacts to psychiatric or somatic hospitals. Patient costs included out-of-pocket medical expenses, including expenses for both medicine and primary care services. Municipality costs included nurse care, daily care, and domestic help at home. Information on each participant’s use of health care services and prices for public services was retrieved from the Danish Primary National Health Insurance Service Register, the Danish National Patient Registry, and the Danish National Prescription Registry [40,41,46]. Information regarding municipality costs was collected based on the Treatment Inventory of Costs in Psychiatric Patients questionnaire [47], which was administered to all participants at baseline. Baseline costs were calculated for resource use 12 months before the start of the study until the baseline date.

Information on intervention costs was provided by the key personnel at a psychiatric outpatient facility. The intervention costs of the app were estimated as the capital and operating costs. Capital costs included the one-time cost of starting and establishing the mobile diary app program and the education of therapists on how to use the program’s software. Operating costs included costs, such as program licenses. Startup establishment and program license costs were estimated as a range due to price instability of new devices, especially if the device is new in the market [48,49]. The pricing was estimated in collaboration with the software company and public purchasers, with an expected discount if the software were to be implemented in Denmark. The license fee was allocated to an estimated number of patients per therapist. The costs of educating the therapists were estimated using the time allocated for training and the average effective hourly wages for the therapists. The wages were based on information from Statistics Denmark on the national average gross wages corresponding to the therapists’ occupation [50]. Capital costs were annuitized over 3 years, with a discount rate of 4% per year [51,52]. Subsequently, the costs were allocated to an estimated number of patients per therapist per year and were included in the analysis as annual costs. The relevant intervention costs in the paper group included the price of paper and the printing of diaries for 12 months. The choice between the app and paper was assumed to not influence the overhead costs. See Multimedia Appendix 1 [43,44,48-53] for further information on cost estimation.

### Statistical Analysis

To account for missing data, multiple imputation was performed, which was applicable because the missing data were assumed to be missing at random [54,55]. The consequences of borderline severity and suicide behavior are shown as nonimputed estimates due to a high number of missing values at both baseline (17 and 20 missing values, respectively) and follow-up (20 and 17 missing values, respectively). The analyses were conducted using an intention-to-treat analysis. See Multimedia Appendix 2 [54] for a summary of the missing data and a full description of the imputation model.

Data are reported either as the number of participants in each group and percentages, median, and IQR, or as means and SE. Incremental health-related outcomes were estimated as both unadjusted and adjusted outcomes based on the regression analysis. The health-related outcomes were adjusted for age, sex, relationship status, education level, baseline scores (utility score, depression score, suicide score, and borderline score), and baseline costs to control for baseline differences [38]. For consequences related to the health care sector and treatment compliance, *P* values were calculated using the Fisher exact test for categorical variables and Student *t* test (2-tailed) for continuous variables [56]. *Statistical* significance was set at a *P* value <.05. Statistical analyses were performed using the STATA (version 16.1, StataCorp).

### Ethics and Consent to Participate

The trial was registered at ClinicalTrials.gov (identifier NCT03191565) and was performed in accordance with the Declaration of Helsinki and approved by the Danish Ethics Committee in the Region of Southern Denmark (Registration number S-20160085). Participants were recruited between June 2017 and December 2018. All participants signed an informed consent form before participating in the study.

### Data Sharing Statements

The data sets generated and analyzed during this study are available from the corresponding author upon reasonable request.

## Results

### Overview

In total, 79 participants were enrolled in the trial, of whom 78 were eligible for inclusion in the analysis. Participants in both groups were predominantly women, with a mean age of 29 years (Table 1).

**Table 1:** Characteristics of the participants at baseline.

Study population			Mobile diary app (N=42)		Paper diary cards (N=36)
Age (years), median (IQR)			26.4 (22.2-32.8)		27.2 (22.3-34.6)
Sex (female), n (%)			>37 (≥88)		>31 (≥86)
**Cohabiting status, n (%)**					
	Living with somebody	14 (33)		18 (50)	
	Living alone	28 (67)		18 (50)	
**Education, n (%)**					
	Education <3 years^a^	>37 (≥88)		27 (75)	
	Education ≥3 years^b^	<5 (≤12)		9 (25)	
**Employment, n (%)**					
	In employment	9 (21)		9 (25)	
	Not in employment	33 (79)		27 (75)	
**Costs^c^ (US $), mean (SE)**					
	Health care costs	17,200.35 (3351.31)		15,684.14 (2800.31)	
	Municipality costs	7785.36 (5680.32)		250.08 (140.15)	
	Patient costs	303.66 (49.47)		327.02 (48.09)	
	Total costs	25,289.37 (6918.34)		16,378.66 (2805.81)	

^a^Participants with highest education of public school, high school, further education <3 years and apprenticeship.

^b^Participants with further education ≥3 years.

^c^Annual cost the year before the study start date.

### Consequences

Participants in both groups experienced an increase in utility scores from baseline to the 12-month follow-up (Table 2). The adjusted QALY gain difference between the app group and the paper group was −0.054 (SE 0.03; *P*<.001), indicating a smaller QALY gain in the app group. The unadjusted QALY gain difference was close to the adjusted estimate. Both groups showed improvements in the EQ-5D domains, except for domain 1 (mobility) in the app group and domain 2 (self-care) in the paper group. The improvement in domain 5 (anxiety and depression) was comparable between the 2 groups.

**Table 2:** Quantitative consequences of the mobile diary app and paper-based diary cards.

Health-related outcomes				Mobile diary app (N=42)		Paper diary cards (N=36)		Unadjusted between-group difference			*P* value			Adjusted between-group difference			*P* value	
**EQ-5D^a^ levels, utility and QALY^b^, mean (SE)^c^**																		
	**Baseline**								—^d^			—			—			—
		Domain 1- Mobility	1.86 (0.15)		1.89 (0.17)													
		Domain 2—Self-care	1.52 (0.14)		1.31 (0.10)													
		Domain 3—Usual activities	2.86 (0.14)		2.67 (0.15)													
		Domain 4—Pain or discomfort	2.93 (0.16)		2.58 (0.21)													
		Domain 5—Anxiety or depression	3.17 (0.17)		3.11 (0.15)													
		Utility score	0.45 (0.05)		0.51 (0.05)													
	**Follow-up**								—			—			—			—
		Domain 1-Mobility	2.2 (0.22)		1.57 (0.13)													
		Domain 2-Self-care	1.37 (0.13)		1.41 (0.14)													
		Domain 3-Usual activities	2.56 (0.19)		1.95 (0.17)													
		Domain 4-Pain or discomfort	2.66 (0.17)		2.30 (0.15)													
		Domain 5-Anxiety or depression	2.41 (0.17)		2.33 (0.18)													
		Utility score	0.6 (0.06)		0.74 (0.03)													
	QALY gain			0.078 (0.03)		0.115 (0.03)		-0.037 (0.04)			.38			-0.054 (0.03)			<.001	
**Borderline severity, mean (SE)^e^**																		
	Baseline			18.6 (1.22)		19.5 (1.08)		—						—			—	
	Follow-up			8.82 (1.16)		8.83 (1.45)		—						—			—	
	Change within the group			9.85 (1.28)		10.67 (1.39)		−0.81 (1.93)			.67			−0.67 (1.73)			.004	
**Depression severity, mean (SE)^c^**																		
	Baseline			17.52 (0.80)		16.11 (0.88)		—			—			—			—	
	Follow-up			12.34 (1.03)		10.47 (1.3)		—			—			—			—	
	Change within the group			5.18 (1.01)		5.64 (1.38)		−0.46 (1.67)			.78			−1.11 (1.57)			.04	
**Suicidal behavior, mean (SE)^e^**																		
	Baseline			11.60 (0.75)		11.96 (0.88)		—			—			—			—	
	Follow-up			8.86 (0.58)		9.38 (0.62)		—			—			—			—	
	Change within the group			2.79 (0.59)		2.76 (0.72)		0.03 (0.93)			.98			0.47 (0.73)			<.001	

^a^EQ-5D: EuroQol 5-Dimensions.

^b^QALY: quality-adjusted life year.

^c^Imputed data set.

^d^Empty cells concern descriptive measures not relevant for statistical testing.

^e^Variables with missing values.

Participants in both groups had decreased borderline severity, depressive severity, and suicidal behavior during the 12-month follow-up. The adjusted difference in the change within the group in depression severity between the app group and the paper group was −1.11 (SE 1.57; *P*=.04), indicating that participants in the app group had a smaller decrease in depression severity. The unadjusted differences showed a similar result. In addition, participants in the app group had a smaller decrease in borderline severity but a greater decrease in suicidal behavior. These results should be interpreted with reservations due to a high number of missing values at both baseline and at follow-up.

The use of health care sector services was similar across both the periods and the groups (difference: psychiatric hospitalization <5 and <5; general practice −1.32, SE 3.68 and 2.02, SE 3.19), indicating that registering in the app compared with on paper does not affect the resource use in the health care sector (Table 3).

The paper group had an insignificantly higher number of participants dropping out before the start of treatment compared with the app group (*P*=.07). Of those starting treatment, participants in the app group were more persistent in adhering to treatment and had an average of 37.1 (SE 27.55) more days of treatment than the paper group. Participants in the app group registered 3.16 (SE 5.10) more skills per week compared with participants in the paper group. Therapists and researchers estimated that the app was time saving for the patient, approximately 1 minute on each diary entry. They further pointed out a potential utility gain for patients from the possibility of choosing according to preferences between registering in an app or on paper diary cards.

**Table 3:** Quantitative consequences of the mobile diary app and paper-based diary cards.

Health-related outcomes				Mobile diary app (n=42)		Paper diary cards (n=36)		Between-group difference		*P* value
**Health care sector contacts**										
**Participants hospitalized in a psychiatric hospital, n (%)**										
	30 days before baseline			<5 (≤12)		<5 (≤14)		≤5		>.99
	30 days after baseline			<5 (≤12)		<5 (≤14)		≤5		>.99
**General practice contacts, mean (SE)**										
	12 months before baseline			19.42 (2.4)		20.75 (2.83)		−1.32 (3.68)		.72
	12 months after baseline			19.02 (2.18)		17 (2.32)		2.02 (3.19)		.53
**Treatment compliance**										
	Participants, who never start treatment, n (%)			<5 (≤12)		7 (19)		≤−2		.07
	Number of treatment days for participants starting treatment, mean (SE)			265.93 (18.64)		228.83 (19.64)		37.1 (27.55)		.18
**Skill recordings, mean (SE)**										
	**All participants**									
		Skills recorded per patient	647.93 (150.72)		409.89 (173.99)		238.04 (229)		.30	
	**Participants starting treatment**									
		Skills recorded per patient	680.33 (156.56)		508.83 (212.54)		171.5 (257.9)		.51	
		Per week	15.04 (3.04)		11.88 (4.29)		3.16 (5.10)		.54	
**Time spent on recording**										
	Time spent on completing the diary (minutes)			4		5		−1		—^a^
**System Usability Scale, mean (SD)**										
	Patients (n=16)			81.2 (9.9)		—		—		—
	Therapists (n=23)			61.6 (18.6)		—		—		—

^a^System usability scale is only reported for the mobile diary group.

The System Usability Score was 81.2 (SD 9.9) in patients, but significantly lower at 61.6 (SD 18.6) in therapists (for further information see Marceau et al [22]). In general, patients were satisfied with the mobile solution, whereas the therapists expressed concern regarding the potential for a negative influence on the patient-therapist interaction due to new tasks related to the implementation of the new technology [22]. In addition, reduced flexibility in data collection due to the standard structure of the app was described as a potential disadvantage. Finally, numerous implementation issues were highlighted in the focus groups, including concerns relating to a potential lack of technical skills of both the patient and therapist, and security and storage of the collected data.

Future potential for innovation was considered relevant as electronic data recording can be used to predict outcomes and benefits from changes in treatment strategy (Textbox 1). The mobile diary app could provide therapists with information on the patients alongside treatment, thereby making it possible to define cut-off values for intervening outside sessions. Program optimization and visualization of the entered data could further inform the patients about their progress, potentially increasing the patient’s self-insight and empowerment.

**Innovation potential of the mobile diary app and paper-based diary cards.**

**Innovation potential**

- Real time recording makes it possible to track patients’ symptoms and possibly intervene outside sessions.
- Electronic data from the app can potentially be used for predicting outcomes from treatment.

### Costs

The between-group difference in total costs during the 12-months ranged from US $107.38 to US $322.13 per participant (Table 4).

**Table 4:** Intervention costs (US $) per participant at 12-month follow-up.

Intervention cost	Mobile diary app (US $)	Paper diary cards (US $)	Difference (US $)
Startup cost^a^	9.41-28.44	0	9.41-28.24
License	97.96-293.88	0	97.96-293.88
Education	3.27	0	3.27
Paper and print	0	3.26	3.26
Total	110.64-325.39	3.26	107.38-322.13

^a^Annuitized over 3 years with a 4% discount rate.

## Discussion

### Principal Findings

To our knowledge, this study is the first to evaluate the costs and consequences of a mobile diary app for patients with BPD. Our findings suggest that using a mobile app as an adjunct to DBT comes with a high acceptance of patients, and the potential for increased treatment adherence and future innovation from electronic database registrations. We also found, however, that the app was associated with a reduced improvement in patients’ depression and health-related quality of life scores within the first year of treatment. The app led to higher costs but was still a relatively inexpensive intervention.

The economic evaluation was conducted using a cost-consequence approach rather than the traditional cost-utility (ie, cost-per-QALY gained) framework. The cost-consequence approach has been criticized by health economists for not being a normative analysis, as the decision-maker has to choose from her or his own opinion concerning the relative importance of the list of consequences [30]. As the mobile diary app was still in early development, we believe there is a need to investigate a broad range of potential consequences, including intermediate consequences. Indeed, health professionals should be encouraged to engage in the debate about how to evaluate internet-based interventions in mental health. Health economists have concluded that the economic evaluation of devices in health care, in general, represents a number of methodological challenges; in particular, the use of QALY as an outcome may be too narrow to capture all relevant benefits to patients and health care organizations [47]. Specifically, within mental health, the use of QALY and EQ-5D has also been found to be challenging with regard to instrument sensitivity [57].

Participants in both the app and paper groups had a QALY gain, but the gain was significantly larger in the paper group. This result is difficult to explain as the difference in QALY gain between the groups was caused by a worsening in the mobility domain of the EQ-5D-5L among app users (Table 2). There is a risk that patients have interpreted the EQ-5D mobility domain differently, either as a question about physical restrictions or as limitations on mobility due to anxiety or depression. Nevertheless, the results suggest that using mobile apps as adjuncts to DBT might reduce the improvement in quality of life and depression scores within the first year.

There are several limitations to this study, including the small sample size, short follow-up period, and missing data. The missing data on depression severity and utility domains were handled by performing multiple imputations and were assessed as not having an impact on the results [54]. There was a considerable amount of missing data for both borderline severity and suicide behavior, making it infeasible to run the imputation model for these outcomes. Therefore, borderline severity and suicidal behavior were presented as nonimputed data, potentially indicating that these results could be biased [55]. Previously, a moderate correlation has been shown between the EQ-5D utility scores and measures for borderline severity, supporting our findings of a similar improvement in QALY and borderline severity [58].

Even though participants in the app group recorded more skills, this did not contribute to a larger QALY gain in the app group than in the paper group. One possible explanation is that a measurement of an effect like health-related quality of life might not capture the potential effects of the app [59], which is why including other effects is relevant. In this study, potential effects such as new and better information from the patients are highlighted, which is supported by the findings of Lauritsen [60], who concluded that the use of a mobile electronic diary can improve the quality and accuracy of data entered into the diary. A study by Derks et al [61] found that a mobile diary app together with a biosensor was effective in increasing self-awareness in patients with BPD. Similar results were found in this study, as key personnel stated that increased self-insight and empowerment might be a potential advantage linked to self-monitoring. Furthermore, both in this study and in prior studies [62,63], a mobile app has been proven to enhance treatment compliance. Thus, if this study had been conducted as a cost-utility, the aforementioned consequences would not have been included, meaning the conclusion might have been quite different.

In this study, concerns regarding the app were primarily from therapists who worried that the app would negatively affect their interaction with the patient. In contrast, findings by Austin et al [64] suggests that a mobile app integrated into DBT was useful for patients with BPD in building an alliance with their therapist and that it promoted collaboration between therapist and patient. However, it is important to state that participants who enrolled in this study and the study by Austin et al [64] might have been advocates for technology since they knew the new and alternative treatment would be a mobile app, which could explain why patients were pleased with the app and why it was primarily therapists who had concerns. Thus, the impact of a mobile diary app on the therapist-patient relationship should be explored in future research.

### Conclusions

In conclusion, this study suggests that using a mobile diary app as an adjunct to DBT has positive as well as negative consequences for patients and, on average, will lead to higher costs than paper-based diary cards. The use of a mobile diary app in the treatment of patients with BPD is still a relatively new field of research, and further investigation in this area is essential.

## Appendix

Multimedia Appendix 1 Detailed information on cost estimates in the economic analysis.

Multimedia Appendix 2 A detailed description of the used approach for multiple imputation.

## Abbreviations

BPD: borderline personality disorder
DBT: dialectical behavior therapy
EQ-5D-5L: EuroQol 5-Dimensions 5-Levels
QALY: quality-adjusted life year
