# Supplementary material for: Mobile Diary App Versus Paper-Based Diary Cards for Patients With Borderline Personality Disorder: Economic Evaluation
Source: J Med Internet Res. 2021 Nov 11;23(11):e28874. doi: 10.2196/28874 (PMC8663638; doi:10.2196/28874)
Supplement: Multimedia Appendix 1 [file jmir_v23i11e28874_app1.doc]

**Appendix 1**

Detailed information on cost estimates.

| **Assumption** | **Estimate** | **Elaboration** | **Reference** |
| --- | --- | --- | --- |
| Expected number of patients per therapist | 12 patients | Expected number of patients per therapist per month | Key personnel |
| Conversion rate from DKK to USS dollars | DKK634,0  per $100 | From 23 September 2021 | [1] |
| Index level from net  price index | 2016 = 100.5  2017 = 101.7  2018 = 102.6  2019 = 103.5 | Primary index level used to adjust costs data to the price level in 2019.  Calculation: (price*new index)/ old index | [2] |
| **Annuitizing one-time startup costs** | | | |
| Costs items annuitized |  | Establishment of the mobile diary app program and education of therapists. |  |
| App lifetime and education | 3 years | Applied lifetime for a technology or education of the therapists. | [3] |
| Discount rate | 4% | Applied discount rate for annuitizing | [4] |
| Annuity factor | 2.77509 | Annuity factor when lifetime 3 years and discount rate 4% | [5] |
| **Intervention costs** | | | |
| Establishment of the mobile diary app program | $310,9-932,8 | Estimated as a range due to the price instability of a new product[6,7].  Annuitized and allocated to an expected number of patients per therapist. | Program development company and public purchasers |
| Education of therapists |  | Calculated based on time allocated for education and a combined average effective hourly wage.  Annuitized and allocated to an expected number of patients per therapist. |  |
| Time allocated for education | 1 hour x 2 per therapist | Therapists would have 1 hour x 2 to learn the program by themselves. |  |
| Average effective hourly wage for psychologist | $58,7 | Based on statistikbanken.dk – LONS20. Earnings per performed hour. 2634 Work within psychology. Sector: All. Year: 2019. | [8] |
| Average effective hourly wage for nurse | $50,4 | Based on statistikbanken.dk – LONS20. Earnings per performed hour. 2221 Nursing work. Sector: All. Year: 2019. | [8] |
| Combined average effective hourly wage | $54,5 | Psychologists and nurses primarily use the mobile diary app, meaning a physician wage is not included in the combined wage. The estimation is based on a team consisting of an equal distribution of psychologists and nurses as therapists. The average wages for the two professions were combined and divided by two to estimate a combined average wage. |  |
| Program license | $96,8-291,8 per therapist a month | Use of the program, support, and server space.  Allocated to an expected number of patients per therapist. | Program development company and public purchasers |
| **Municipality costs** | | | |
| Home care |  | Nurse care, daily care and domestic help. Estimated from average effective hourly wages for the different services. Service use estimates based on the Treatment Inventory of Costs in Psychiatric Patients questionnaire. |  |
| Average effective hourly wage for daily care and domestic help | $39,5 | Based on statistikbanken.dk – LONS20. Earnings per performed hour. 5322 Social and health care work in private homes. Sector: Municipality. Year: 2019. | [8] |
| Average effective hourly wage for nurse working in municipality | $51,8 | Based on statistikbanken.dk – LONS20. Earnings per performed hour. 2221 Nursing work. Sector: Municipality. Year: 2019. | [8] |

**References**

1. EUROinvestor. Valutakurser [Exchange rates] [Internet]. 2019 [cited 2020 Jun 25]. Available from: https://www.valutakurser.dk/

2. Statistics Denmark. Nettoprisindeks [Net price index] [Internet]. 2020 [cited 2020 Nov 4]. Available from: https://www.dst.dk/da/Statistik/emner/priser-og-forbrug/forbrugerpriser/nettoprisindeks

3. Agency for Modernisation - Ministry of Finance. Levetider [Service lives] [Internet]. [cited 2020 Jun 18]. Available from: https://oes.dk/oekonomi/oeav/regnskabsregler/generelle-bogfoeringsbestemmelser/levetider/

4. Ministry of Finance. Den samfundsøkonomiske diskonteringsrente [The economic discount rate]. 2018.

5. Ehlers LH, Vestergaard AS. Costing in health economic evaluation. Aalborg University Press; 2019.

6. Kirisits A, Redekop WK. The Economic Evaluation of Medical Devices. Appl Health Econ Health Policy [Internet] 2013 Feb 4;11(1):15–26. [doi: 10.1007/s40258-012-0006-9]

7. Drummond M, Tarricone R, Torbica A. Economic Evaluation of Medical Devices. Oxford Res Encycl Econ Financ [Internet] Oxford University Press; 2018. [doi: 10.1093/acrefore/9780190625979.013.105]

8. Statistics Denmark. Statistikbanken - LONS20: Løn efter arbejdsfunktion, sektor, aflønningsform, lønmodtagergruppe, lønkomponenter og køn [Statistikbanken - LONS20: Wages estimated based on job function, sector, payment form, employment group, wage component and gender] [Internet]. 2018 [cited 2020 Jun 10]. Available from: https://www.statistikbanken.dk/statbank5a/default.asp?w=1536
