# Supplementary material for: Mobile Diary App Versus Paper-Based Diary Cards for Patients With Borderline Personality Disorder: Economic Evaluation
Source: J Med Internet Res. 2021 Nov 11;23(11):e28874. doi: 10.2196/28874 (PMC8663638; doi:10.2196/28874)
Supplement: Multimedia Appendix 2 [file jmir_v23i11e28874_app2.doc]

**Appendix 2**

A detailed description of the used approach for multiple imputation in accordance with methods described by Faria et al.[1].

At baseline, all participants (n=78) completed the Patient Health Questionnaire-9 (PHQ-9), the EuroQol 5-Dimensions 5-Levels (EQ-5D-5L) questionnaire and the Treatment Inventory of Costs in Psychiatric Patients questionnaire (TiC-P). Of the 78 participants included in the analysis, 61 participants (78.2% (app=35, paper-based=26) had information from the Zanarini Rating Scale for Borderline Personality Disorder (ZAN BPD) and 58 participants (74.4% (app=33, paper-based=25) filled in the Suicide Behaviors Questionnaire (SBQ) at baseline. At the 12-month follow-up, 61 participants completed the PHQ-9, EQ-5D-5L questionnaire and SBQ (78.2% (app n=35, paper-based n=26)) and 58 participants (74.4% (app=34, paper-based=24) had assessments based on ZAN BPD.

All costs were complete for all participants.

After a visual inspection of the missingness pattern, it was assumed that the data missing was missing at random (MAR). Therefore, multiple imputations were performed.

The imputation model used chained equations for both consequences and costs. The EQ-5D-5L components were imputed at a single component level, whereas the missing values for PHQ-9 was imputed at a summary score level. Municipality costs were imputed as an aggregated cost, not specifying whether the cost was related to domestic help, nurse care or daily care. The imputation was performed using the command *mi imputed chained (pmm, knn (x))* in STATA version 16. The imputation used predictive mean matching with a knn=five, and was set to generate 40 datasets. The imputation model included sociodemographic variables such as age, gender, working status, educational level, and relationship status. Moreover, the model included EQ-5D-5L components, PHQ-9 score and SBQ score at both baseline and follow-up. Baseline municipality costs and total costs excluding municipality costs one year before the baseline date was also included. Lastly, total number of treatment days and total number of registered skills was included. The imputation model was run separately for the app group and the paper group.

**References**

1. Faria R, Gomes M, Epstein D, White IR. A Guide to Handling Missing Data in Cost-Effectiveness Analysis Conducted Within Randomised Controlled Trials. Pharmacoeconomics [Internet] 2014 Dec 29;32(12):1157–1170. [doi: 10.1007/s40273-014-0193-3]
